# Self-assembled GA-Repeated Peptides as a Biomolecular Scaffold for Biosensing with MoS_2_ Electrochemical Transistors

**DOI:** 10.1021/acsami.2c23227

**Published:** 2023-03-09

**Authors:** Hironaga Noguchi, Yoshiki Nakamura, Sayaka Tezuka, Takakazu Seki, Kazuki Yatsu, Takuma Narimatsu, Yasuaki Nakata, Yuhei Hayamizu

**Affiliations:** † Department of Materials Science and Engineering, School of Materials and Chemical Technology, Tokyo Institute of Technology, Tokyo 152-8550, Japan; ‡ Department of Frontier Materials Chemistry, Faculty of Science and Technology, Hirosaki University, 3 Bunkyo-cho, Hirosaki, Aomori 036-8561, Japan

**Keywords:** MoS_2_, biosensor, peptides, self-assembly, molecular scaffold, FET

## Abstract

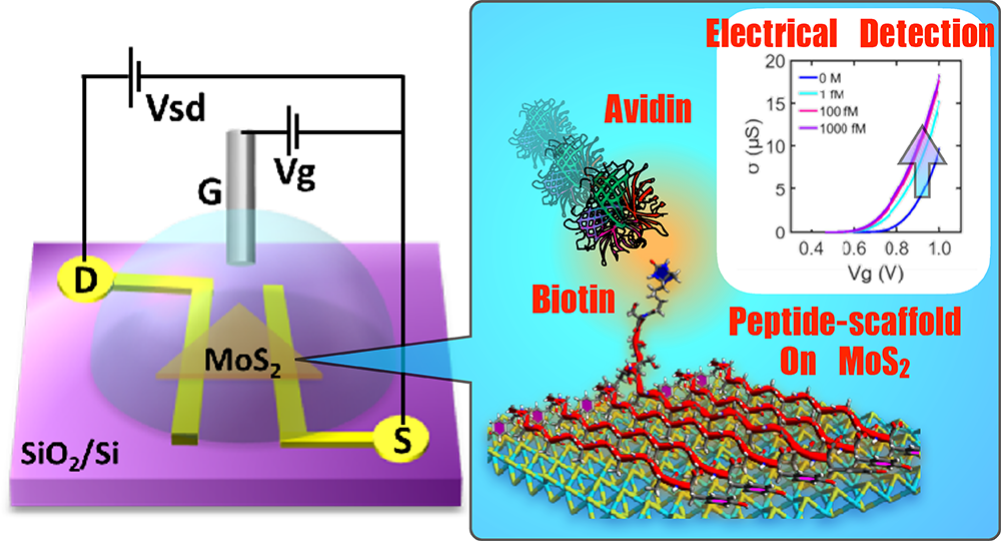

Biosensors with two-dimensional materials have gained wide interest due to their high sensitivity. Among them, single-layer MoS_2_ has become a new class of biosensing platform owing to its semiconducting property. Immobilization of bioprobes directly onto the MoS_2_ surface with chemical bonding or random physisorption has been widely studied. However, these approaches potentially cause a reduction of conductivity and sensitivity of the biosensor. In this work, we designed peptides that spontaneously align into monomolecular-thick nanostructures on electrochemical MoS_2_ transistors in a non-covalent fashion and act as a biomolecular scaffold for efficient biosensing. These peptides consist of repeated domains of glycine and alanine in the sequence and form self-assembled structures with sixfold symmetry templated by the lattice of MoS_2_. We investigated electronic interactions of self-assembled peptides with MoS_2_ by designing their amino acid sequence with charged amino acids at both ends. Charged amino acids in the sequence showed a correlation with the electrical properties of single-layer MoS_2_, where negatively charged peptides caused a shift of threshold voltage in MoS_2_ transistors and neutral and positively charged peptides had no significant effect on the threshold voltage. The transconductance of transistors had no decrease due to the self-assembled peptides, indicating that aligned peptides can act as a biomolecular scaffold without degrading the intrinsic electronic properties for biosensing. We also investigated the impact of peptides on the photoluminescence (PL) of single-layer MoS_2_ and found that the PL intensity changed sensitively depending on the amino acid sequence of peptides. Finally, we demonstrated a femtomolar-level sensitivity of biosensing using biotinylated peptides to detect streptavidin.

## 1 Introduction

Biosensors have offered various applications such as environmental monitoring^1^ as well as medical diagnosis.^2^ Electrical detection of the target molecules with transistor-based biosensors is currently a ubiquitous mechanism for biosensing. To enhance the transistor-based biosensors’ sensitivity, nanomaterials such as Si nanowires and carbon nanotubes are often used for the detection-channel part of the transistor because of their high specific surface area.^3−6^ More recently, biosensors with two-dimensional (2D) nanomaterials represented by graphene and MoS_2_ have demonstrated a high sensitivity to a variety of target molecules.^7−15^ While graphene is semi-metallic, MoS_2_ is a new member of the 2D materials with a semiconducting property, allowing for higher electrical sensitivity for biosensing, in principle, than graphene biosensors.^16^

Surface immobilization of bioprobes onto MoS_2_ biosensors is a key step in realizing a specific sensitivity to target molecules. So far, the following methods have been reported: (1) coating of a dielectric layer, e.g., a HfO_2_ layer with a high κ, on top of the MoS_2_ transistor, and subsequent immobilization of the bioprobes on top. This method aims for an enhancement of capacitance at the interface and for immobilization of bioprobes with covalent chemistry.^16,17^ A drawback of this method is the relatively large distance between bioprobes and MoS_2_, which causes a decrease in sensitivity. (2) Direct immobilization of the bioprobes on MoS_2_ with a covalent bonding.^18^ While the binding of the probes on the surface is stable, covalent chemistry can degrade the intrinsic electrical properties of MoS_2_. (3) Direct immobilization of the bioprobes on MoS_2_ with physisorption.^19,20^ Although this process can relatively easily immobilize bioprobes, their orientation can be randomized, often resulting in the low activity of the bioprobes. In the case of (2) and (3), the thickness of the molecular scaffold for the bioprobes needs to be sufficiently thinner than the Debye length to maintain the potential sensitivity of the biosensor.

As an alternative way, self-assembled peptides as a molecular scaffold have offered a non-covalent way to immobilize bioprobes onto 2D materials. In recent years, peptides that spontaneously form monomolecular-thick and organized structures on two-dimensional materials such as graphite,^21−25^ graphene,^26−28^ and MoS_2_^27,29−32^ have been reported. The structures exhibited a clear symmetric feature reflecting the underlying lattice of 2D materials, indicating structural matching between the periodic structure of supramolecular peptides and the lattice of 2D materials. The aligned structures have been proven to be stable under an aqueous solution and even under an applied electrical bias.^33^ Furthermore, biosensing has been demonstrated by immobilizing bioprobes supported by self-assembled peptides on graphene/graphite surfaces in a controlled manner.^33,34^ Because of increasing interest in the application of MoS_2_ for biosensing,^11,12,16^ it is essential to unravel the electronic interaction of self-assembled peptides and MoS_2_ and their ability as a molecular scaffold for biosensing.

In this work, we designed a series of new self-assembled peptides with different net charges and utilized electrochemical field-effect transistors (FETs) of single-layer MoS_2_ grown by chemical vapor deposition (CVD)^35,36^ to control electrical interactions of peptides with MoS_2_ and characterized the conductivity of MoS_2_ FETs with the peptide scaffold. Atomic force microscopy (AFM) revealed that these self-assembled peptides showed a high coverage and highly oriented nanostructures with sixfold symmetry on MoS_2_. The thickness of the peptide structures was ∼1 nm, which is sufficiently smaller than the Debye length of >2 nm for a typical buffer solution or physiological solution. Electrical measurements of MoS_2_ FETs showed that the transistor mobility was not reduced by peptide self-assembly on the surface, manifesting that these peptides can work as molecular scaffolds for biosensing without degrading the intrinsic electronic properties of MoS_2_. The threshold voltage of the FET was affected depending on the types of peptides and their self-assembly condition. The photoluminescence (PL) of single-layer MoS_2_ was also characterized before and after the peptide self-assembly, and it was found that the PL intensity was increased or decreased depending on the peptides, indicating that the charged amino acid in the peptide sequences sensitively interacted with MoS_2_. Furthermore, we demonstrated a proof of concept for biosensing with biotinylated peptides immobilized on MoS_2_ FET via co-assembly and successfully detected the binding event of streptavidin on the bioprobes with high sensitivity.

## 2 Materials and Methods

### 2.1 Peptide Synthesis

Peptides were synthesized by a solid-phase peptide synthesis method.^37^ Rink Amide MBHA resin as a solid support in peptide synthesis was swelled in *N*,*N*-dimethylformamide (DMF) overnight. Then, the resin was deprotected using a 20% piperidine/DMF solution and washed with DMF. For peptide extension, we utilized 1-hydroxybenzotriazole monohydrate, *O*-(benzotriazol-1-yl)-*N*,*N*,*N*′,*N*′-tetramethyluronium hexafluorophosphate, and *N*,*N*-diisopropylethylamine as coupling reagents with an amino acid protected by a 9-fluorenylmethyloxycarbonyl group (Fmoc) for the N-terminal. The cycles of deprotection–wash–coupling–wash were repeated. Then, we obtained the resin with target peptides. The target peptides were cleaved from the resin with trifluoroacetic acid/triisopropylsilane aqueous solution and collected with extraction/precipitation processes. The obtained crude peptide powder was purified by reverse-phase HPLC using an acetonitrile/water mobile phase. See the details of the Fmoc chemistry and purification in the Supporting Information.

### 2.2 Synthesis of CVD-Grown Single-Layer MoS_2_ for MoS_2_ FETs and PL Measurement

MoS_2_ was synthesized on a Si wafer with a 270 nm-thick SiO_2_ layer by CVD with a two-zone furnace.^12^ Sulfur (40 mg) and molybdenum dioxide (MoO_2_) powders (10–20 mg) were used as precursors. Sodium chloride (NaCl) powder (2–10 mg) was mixed with MoO_2_ to improve the crystallinity of MoS_2_. Sulfur powder in a ceramic boat was placed in the first zone located upstream. MoO_2_ and NaCl powder in a ceramic boat were placed in the second zone, and the Si wafer was placed on top of the boat of MoO_2_/NaCl powder. The temperatures for the first and second zones were kept at 200 and 800 °C, respectively. During the growth, argon gas was flown as a carrier gas with a flow rate of 100 cc/min.

### 2.3 Preparation of Samples for AFM Measurements

Flakes of multilayer MoS_2_ were prepared by mechanical exfoliation and transferred on a Si wafer with a 270 nm-thick SiO_2_ layer. Peptides were dissolved in 10 mM phosphate buffer (PB) to obtain the desired concentrations. A droplet of 50 μL peptide solution was placed on the sample and was kept in a humid chamber at room temperature for 1 h. After the incubation, the droplet was replaced with DI water to remove the excess peptides and salt, and then, blown off by nitrogen gas. The resulting peptide sample was dried in a vacuum desiccator for ex situ AFM measurement. For in situ AFM measurement, the peptide sample was kept in a wet condition without removing the droplet on the substrate. Note that the previous paper shows that self-assembled peptides do not have a specific affinity or form of molecular surface recognition depending on the number of MoS_2_ layers.^32^ Thus, we utilized both substrates of bulk for AFM measurements and single layers for PL and FET measurements.

### 2.4 AFM Measurements

**Figure 1:**
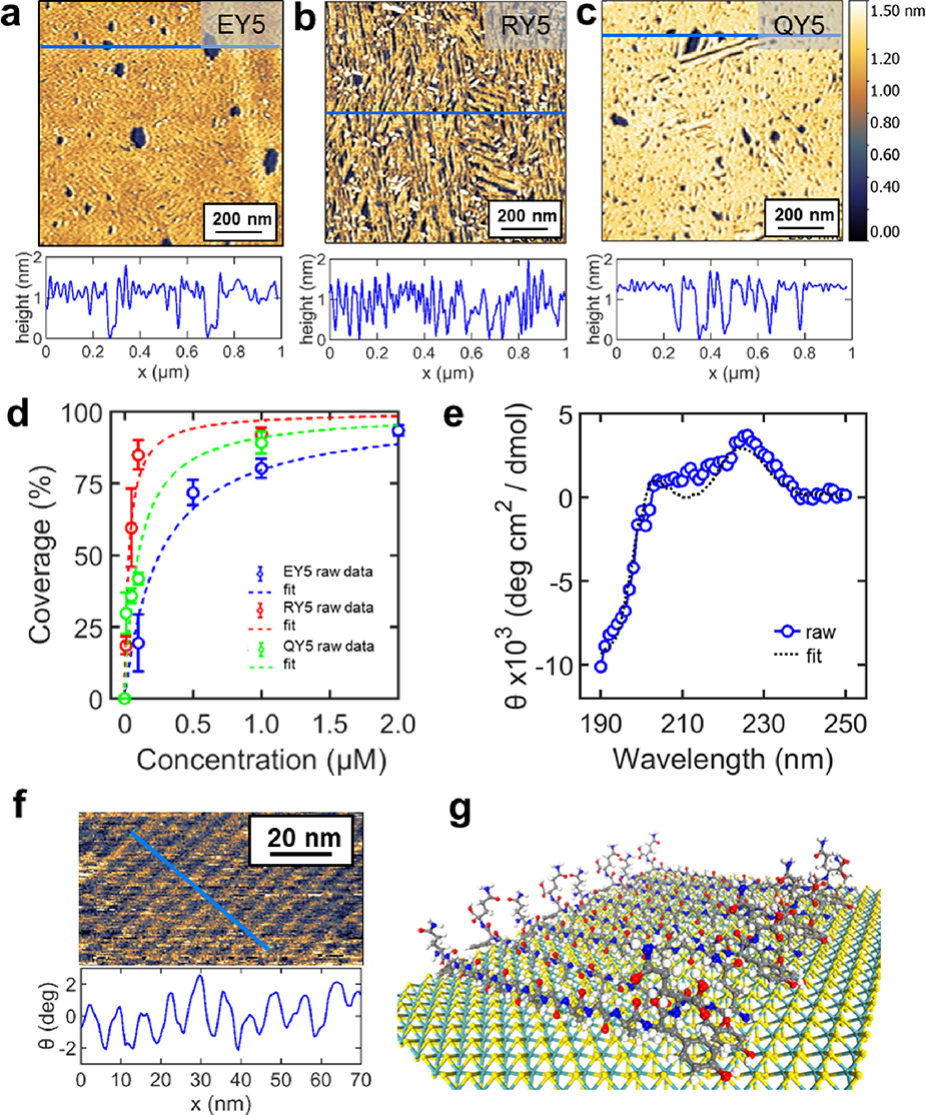
(a–c) AFM topography images and cross-sectional profiles of self-assembled peptides on the MoS_2_ surface for three different peptides, EY5, RY5, and QY5. The cross-sectional profile was obtained at the solid blue lines in the AFM images. (d) Coverage of self-assembled peptides on MoS_2_ as a function of concentrations for the peptide solution. (e) Circular dichroism spectrum of the QY5 peptide at a 50 μM concentration. The fit was generated from BeStSel.^39,40^ (f) In situ AFM phase image of QY5 on MoS_2_ and its cross-sectional profile. (g) Proposed molecular model of self-assembled peptides on the MoS_2_ surface.

The morphology of self-assembled peptide structures was characterized by an AFM (AFM5500, Keysight Technology) with AC mode in the air. It was equipped with a silicon cantilever (OMCL-AC160TS, Olympus, JP) with a resonance frequency of 300 kHz and a spring constant of 26 N/m. The obtained AFM images were processed with Gwyddion (Czech Metrology Institute, CZ). The in situ measurement of peptide self-assembly (Figure 1f) was performed with a cantilever (BL-AC40TS-C2, Olympus, JP) with a resonance frequency of 25 kHz and a spring constant of 0.5 N/m. The measurement was done under a 1 μM peptide solution.

### 2.5 PL Measurement of MoS_2_

The PL of single-layer MoS_2_ was obtained by an inverted microscope (IX73, Olympus) with a spectrometer (Shamrock 193i, Oxford Instruments) equipped with an electron-multiplying charge-coupled device (iXon-Ultra 888 EMCCD, Oxford Instruments). The excitation light of ∼546 nm filtered from the mercury lamp’s white light with a band-pass filter was guided to the samples through a dichroic mirror and 100× objective lens (N.A. = 0.95). The details of the optical setup are shown in the Supporting Information.

### 2.6 Demonstration of FET-Based MoS_2_ Biosensors

The conductivity (σ) of MoS_2_ FETs was derived from the following equation:
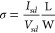
1where *L*, *W*, *I_sd_*, and *V_sd_* denote the channel length, the channel width, source-drain current, and source-drain voltage, respectively. The transconductance *g_m_* was estimated from the ratio of the change in the source-drain current to the change in the gate voltage. A Keithley 4200 was used to measure the electrical conductivity of MoS_2_ FETs. The gate voltage was electrochemically applied using a platinum (Pt) wire as a reference electrode, and 10 mM PB was used as an electrolyte solution. The range of the gate voltage was 0.3–1.0 or 0.3–1.4 V with a *V_sd_* of 30 mV.

## 3 Results and Discussion

We designed peptides based on a previously reported sequence of peptides that has a repeated sequence of glycine–alanine (GA).^33^ The GA sequence has been employed from a pattern of the sequence found in fibroin of silk protein, which forms a stable β-sheet structure. At both the N- and C-terminal ends of the GA-repeated sequence, characteristic amino acids have been introduced to tune their self-assembly and interaction on the surface. For example, YGAGAGAGAGAY (Y5Y) with tyrosine “Y” at both ends has formed stable ordered structures on the MoS_2_ surface.^33^ The ordered structures have remained even after washing with DI water. This demonstration showed that the GA-repeats in the sequence possessed strong hydrogen bonds among the peptides and remained stable in their self-assembled structures on both graphite and MoS_2_ surfaces. The presence of tyrosine in the sequence also facilitated their interaction with the MoS_2_ surface. Furthermore, the self-assembled structures were stable even under electrochemical bias, suggesting that the interpeptide interactions were strong enough against perturbations by electrolytes in the formation of the electrochemical double layer. In this work, three new peptides were designed based on the sequence of the Y5Y peptide (Table 1 and Figure S1). These peptides, named EY5, RY5, and QY5, have glutamic acid “E” (negatively charged), arginine “R” (positively charged), and glutamine “Q” (neutral) at both terminal ends for the control of the electrical interactions of peptides with MoS_2_, respectively.

**Table 1:**
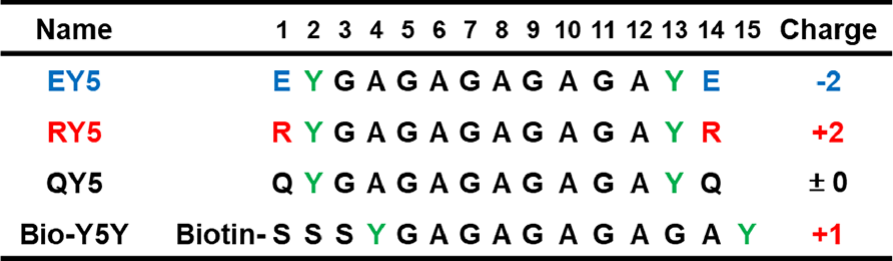
Peptide Sequences and Their Net Charge

Figure 1a–c and Figure S2 show AFM images of self-assembled peptides on the MoS_2_ surface and their cross-sectional height profiles. Each sample was prepared with various concentrations of the peptide solution. All peptides commonly show linear nanostructures at low concentrations (Figure S2). The average thickness of the peptide’s nanostructure was 1.2 nm. As shown in Figure S2, the coverage of peptides on the surface increased as the solution concentration increased. The coverage profile of the peptide over the peptide concentrations was fitted with the Langmuir model (Figure 1d). The binding affinity from the fits were 3.9 ± 0.9 for EY5, 31.7 ± 7.5 for RY5, and 10.0 ± 4.5 μM^–1^ for QY5. While the positively charged peptide RY5 showed the strongest binding affinity, the negatively charged peptide EY5 was the weakest. The variation of the binding affinity (RY5 > QY5 > EY5) probably arises from electrostatic interactions between peptides and the MoS_2_ surface. The affinity of peptides to MoS_2_ surfaces is generally known to be based on hydrophobic interactions.^29^ In addition to the general picture, our findings are consistent with previous reports showing the role of charged amino acids in self-assembly^27^ or binding^31,38^ of peptides on MoS_2_ surfaces. These works showed that the Coulombic interaction affects the interaction with the surface.

We chose QY5 to measure its circular dichroism in DI water. It helps us understand its molecular conformation. The concentration of the peptide solution was 50 μM. Figure 1e shows the obtained spectrum. The fits were in good agreement with the observed spectra. Using the structure analysis tool BeStSel^39,40^ we estimated the composition of secondary structures of peptides. The results show that the peptides’ main structure is antiparallel β-sheets. We also performed in situ AFM to see the periodic structure of self-assembled peptides on MoS_2_ (Figure 1f). The phase image of AFM shows well-aligned linear structures of peptides, where the peptide nanowires have a periodicity of 6.6 nm among each other. A periodic interval of 6.6 nm (for the peptides of 14 amino acid units) is reasonable as the periodic interval for the Y5Y peptide was reported to be ∼5.5 nm (the peptide for 12 amino acid units),^33^ suggesting that these newly designed peptides have the same surface conformation as that for Y5Y. These results indicate that peptides form nanowires on the MoS_2_ surface with a β-sheet structure, and we depicted our proposed molecular model (Figure 1g).

**Figure 2:**
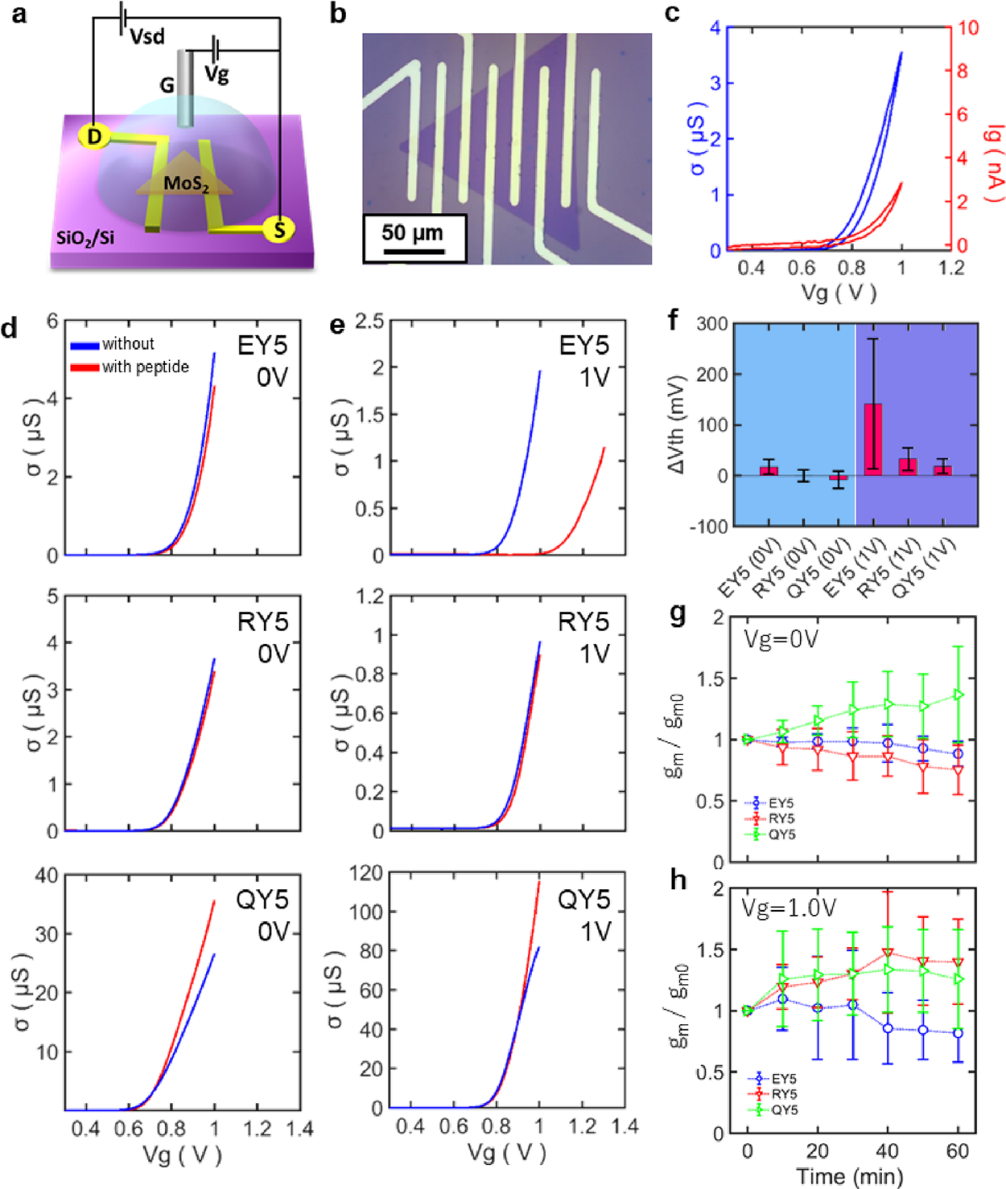
(a) Schematic of MoS_2_ FETs with an electrochemical gating. MoS_2_ was transferred to a pre-patterned source and drain electrodes. (b) Optical micrograph of single-layer MoS_2_ transferred on a substrate with patterned electrodes. (c) Typical conductivity curve of MoS_2_ FET (blue) and leakage current (red) against the applied gate voltage. (d) Conductivity curves of MoS_2_ with and without peptides against the applied gate voltage. (e) Conductivity curves in the case of peptide self-assembly under an applied voltage of 1.0 V. (f) Change of the threshold voltage by self-assembly of peptides on MoS_2_. (g, h) Time-dependent change of transconductance of MoS_2_ FETs during peptide self-assembly under gate voltages of 0 or 1.0 V. The error bars in (f–h) were derived from multiple devices. The details are written in the Supporting Information, Section 3.

Next, we fabricated electrochemically gated MoS_2_ FETs to investigate peptide effects on the electrical conductivity of MoS_2_ (Figure 2a). We synthesized single-layer MoS_2_ by CVD. The CVD-grown MoS_2_ on a Si substrate was transferred to a substrate with prepatterned Au electrodes (Supporting Information Section 3 and Figure S3). The transferred MoS_2_ showed a uniform single domain with a triangular shape without wrinkles (Figure 2b). PL and Raman measurements were carried out to ensure the generation of single-layer MoS_2_ by CVD (Supporting Information Section 3, Figure S4, Supporting Information Section 5, and Figure S6).^41−43^ The transferring technique allows us to prevent contaminations on the MoS_2_ surface due to residues of photo-resist in the lithography process, resulting in a uniform performance of MoS_2_ FETs (Supporting Information Section 4 and Figure S5).

For the characterization of MoS_2_ FETs, we placed 10 mM PB on the FET. A Pt wire was inserted into the PB as a reference electrode. The conductivity (σ) of MoS_2_ FETs with respect to the gate voltage was measured under 10 mM PB. While the source-drain voltage (*V_sd_*) was fixed at 30 mV, the gate voltage (*V_g_*) was swept in the range from 0.3 to 1.0 V cyclically. In Figure 2c, the σ curve of the MoS_2_ FET without peptides showed the threshold voltage (*V_th_*) at around 0.7 V. The hysteresis in the conductivity curve was relatively small. The leakage current between the Pt gate electrode and MoS_2_ (*I_g_*) was below 5 nA (red curve in Figure 2c), which was 100 times smaller than the value of the source-drain current, ensuring the performance of the MoS_2_ FET.

We evaluated the effect of peptides on the conductivity of MoS_2_ FET. We performed conductivity measurements before and after the self-assembly of peptides on MoS_2_ using a Pt wire for applying an electrochemical bias to the MoS_2_ under PB. For the comparison, we utilized three peptides with different net charges, i.e., EY5, RY5, and QY5 (see their sequences in Table 1). For the peptide self-assembly, 1 μM peptide solution diluted with 10 mM PB was used. All the conductivity measurements were performed without drying up the surface of MoS_2_ to prevent undesired side effects due to condensation of salts or deformation of peptide self-assembled structures. We observed the gate response of the conductivity of the peptide-modified MoS_2_ FET (Figure 2d). Surprisingly, none of the three peptides showed a significant change in the conductivity curve after incubation, showing that the threshold voltage *V_th_* and the transconductance *g_m_* were not drastically changed by these peptides. *g_m_* is an essential parameter determining the sensitivity of the biosensor, and the higher *g_m_* means higher transistor mobility or sensitivity. The methodology to derive the *V_th_* and *g_m_* is written in the Supporting Information, Section 4. The preserved transconductance *g_m_* after the peptide self-assembly indicates that the ordered structures of peptides on the surface do not act as scattering points for electrical charge carriers due to their uniform structures. The unchanged threshold voltage *V_th_* also suggests insignificant Coulombic interaction between peptides and MoS_2_.

To further investigate the effect of self-assembled peptides on the σ of MoS_2_ FETs, we incubated peptides under electrochemical potential. Reference (44) reported that self-assembled peptides changed the morphology under electrochemical bias depending on their net charge in the amino acid sequence. We expected that this phenomenon similarly occurs for our peptides. The conformation of the self-assembled peptides under different electrochemical biases possibly differs, resulting in distinct electrical interactions with the MoS_2_ surface unique to each peptide. Based on this working hypothesis, peptide solutions were incubated under an electrochemical bias of 1.0 V, above the threshold voltage of our MoS_2_ FETs. The surface potential under this applied voltage is expected to be negative due to the accumulated electrons in MoS_2_. The σ of MoS_2_ FETs exhibited a different behavior from the one without applying voltage during the peptide incubation (Figure 2e). While the MoS_2_ FET with EY5 showed an increase in the threshold voltage, RY5 and QY5 did not show any change on it.

These observations are summarized in Figure 2f. RY5 and QY5 did not affect the threshold voltage clearly, whereas EY5 caused a non-negligible change of the threshold voltage with more than 100 mV. Interestingly, RY5 and QY5 did not show any change in the threshold voltage, even under the applied voltage. In this sense, RY5 and QY5 are good candidates as molecular scaffolds for biosensing due to their inert nature to the intrinsic electrical properties of MoS_2_. To better understand the effect of peptides on the σ of MoS_2_ FETs, changes of *g_m_* during peptide self-assembly on MoS_2_ FETs are plotted over time for both cases with (Figure 2g) and without (Figure 2h) applying voltage. EY5 showed a decrease in the *g_m_* in the both cases. On the other hand, QY5 did not show any decrease in the *g_m_* but an increase in it. RY5 had an increase in the *g_m_* with applying voltage during the peptide self-assembly but showed a decrease without applying voltage. From above, we found that QY5 did not show any change in *V_th_* nor a decrease in *g_m_*, thus suggesting that QY5 can be the most appropriate peptide as a molecular scaffold among our peptides. Note that to deduce Δ*V_th_* and *g_m_* while minimizing the variations of the MoS_2_ FETs’ electronic properties, we characterized at least three devices for each peptide and display the averaged values in Figure 2f–h (see the details in the Supporting Information).

**Figure 3:**
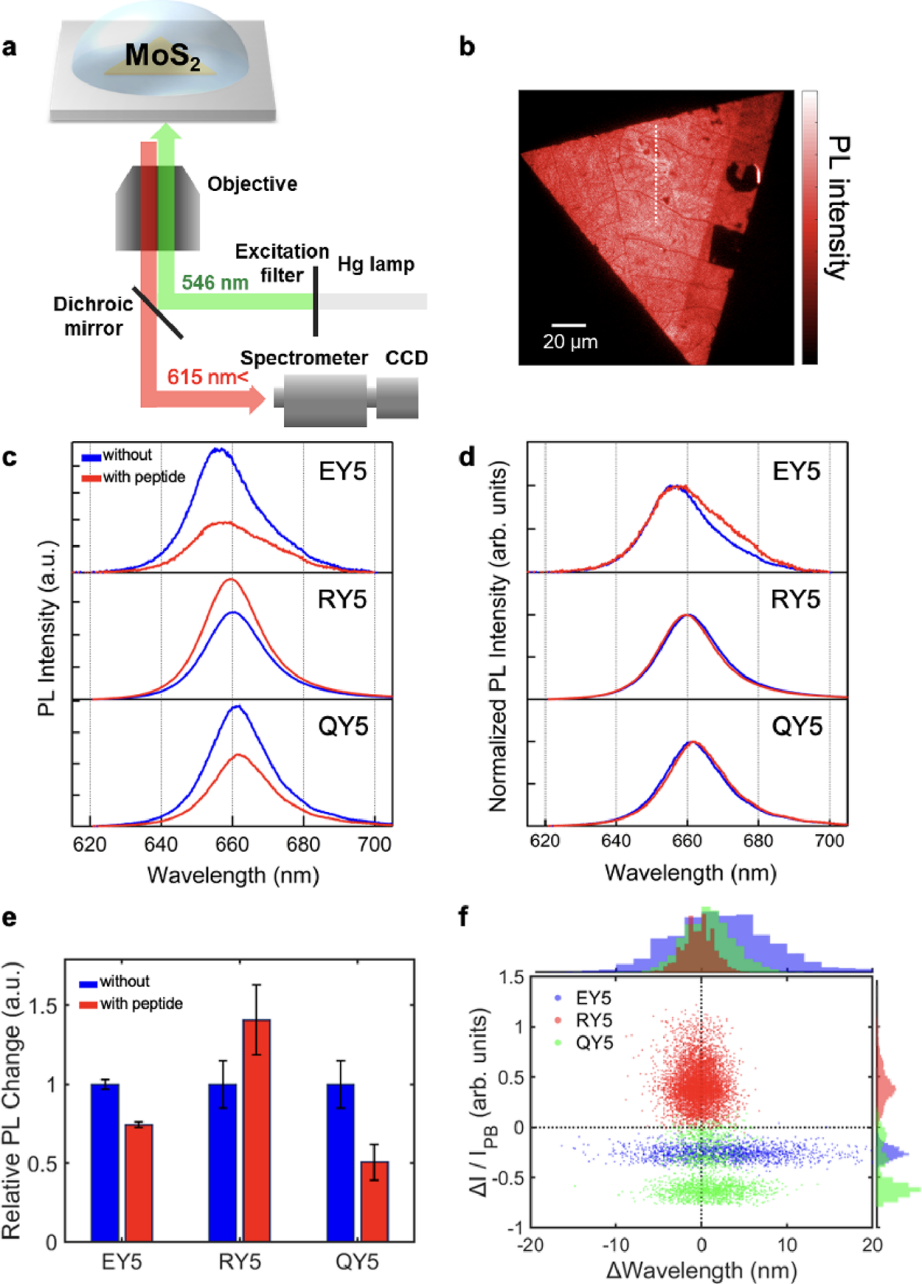
(a) Schematic illustration of the PL experimental setup. (b) Typical PL image of single-layer MoS_2_ after transferring on a coverslip. The white dashed line shows the area used for the spectral measurements. (c) Typical PL spectra of single-layer MoS_2_ before and after self-assembly of peptides. (d) Normalized PL spectra of (c). (e) Changes of the PL peak intensities by peptides. The error bars were obtained by averaging multiple data points with more than four crystals of MoS_2_. (f) Scatter plot of changes in wavelength and intensity before and after peptide self-assembly (see the details of the procedure in the Supporting Information, Section 5).

We also testified the modulation of the optical properties of single-layer MoS_2_ by the peptides’ self-assembly. CVD-grown MoS_2_ was transferred onto a cover glass, and the PL image of MoS_2_ and the corresponding PL spectra were obtained from the sample (Figure 3a). The PL image showed multiple cracks, which were probably created during the transferring process (Figure 3b). Despite the existence of these cracks, the obtained PL spectra showed a homogeneous PL intensity in all the places, indicating that the quality of the synthesized MoS_2_ was uniform. Then, we performed the peptide self-assembly under 50 μL of 500 nM aqueous peptide solutions prepared with 10 mM PB for 1 h, where this concentration of peptides allows us to have high coverage of self-assembled peptides on the MoS_2_ surface (Figure S2).

PL spectra of the peptide-modified MoS_2_ differ significantly with the types of the peptides (Figure 3c). While RY5 had an increase in the peak intensity, EY5 and QY5 showed a decrease in the intensity. Interestingly, the spectral shape of PL peaks showed no change in the case of peptide self-assembly of RY5 and QY5 (Figure 3d), suggesting that the population ratio of excitons and trions in MoS_2_ did not change by the peptides’ nanostructures,^12^ thereby indicating that the electron density in MoS_2_ was not affected by the presence of peptides on the surface. This interpretation is also supported by the unaffected threshold voltages in FETs after peptide self-assembly (Figure 2f). In the case of EY5, the PL spectral shape slightly changed after peptide self-assembly, and a shoulder appeared at the longer wavelength side, indicating that contribution of excitons in the PL decreased compared with that of trions. Summarizing the above, we found that the relative PL changes with the modification of EY5, RY5, and QY5 peptides on MoS_2_ were 0.74, 1.41, and 0.51, respectively (Figure 3e).

We further analyzed the spectral intensity change at various locations on multiple single-layer MoS_2_ crystals for each peptide. The details of the analysis are shown in the Supporting Information, Section 5 (Figure S6). The scatter plot in Figure 3f shows changes in PL intensity and peak position after peptide self-assembly on single-layer MoS_2_. Each point represents a local PL property of MoS_2_, where blue, red, and green dots indicate the values of MoS_2_ modulated by EY5, RY5, and QY5, respectively. First, the peak position did not change for all the peptides on average. RY5 and QY5 showed a narrow distribution in the peak position shift, whereas EY5 showed a broad distribution. The negative charge of EY5 may interact specifically with MoS_2_, resulting in a larger shift of the peak position than those for the other peptides. In terms of the PL intensity, RY5 showed an increase, whereas EY5 and QY5 showed a decrease.

Different from the observation in the conductivity measurements of the MoS_2_ FET, where the threshold voltage did not change by peptide self-assembly for all three peptides, the PL is more sensitive to the adsorbates or vicinity environmental conditions. The constant threshold voltage in the conductivity measurements indicated that the charge carrier density in MoS_2_ was not modulated largely by peptides. Thus, we assume that the change in the PL intensity originated from the quantum yield modulated by the presence of peptides on the MoS_2_ surface. The way of modulation can depend on the peptide sequence with a different net charge.

It is also worth noting that QY5 exhibited three peaks in its distribution of the PL intensity (Figure 3f). This indicates that there were multiple ways for QY5 to interact with MoS_2_, possibly due to the fact that the atomic structure of MoS_2_ is in multiple states, such as edges and defects. In general, the peak shift of the PL spectra and enhancement/reduction of PL intensity can be traced to the change of the local dielectric constant or the strain of MoS_2_.^45,46^ To clarify the whole mechanism of the PL modulation, further understanding from the viewpoint of quantum mechanics is needed by taking into account the structure of water molecules and peptides at the interface as the surface nanostructure and the water distribution, which critically affects the local dielectric environment and thus local electric environment.^47,48^

**Figure 4:**
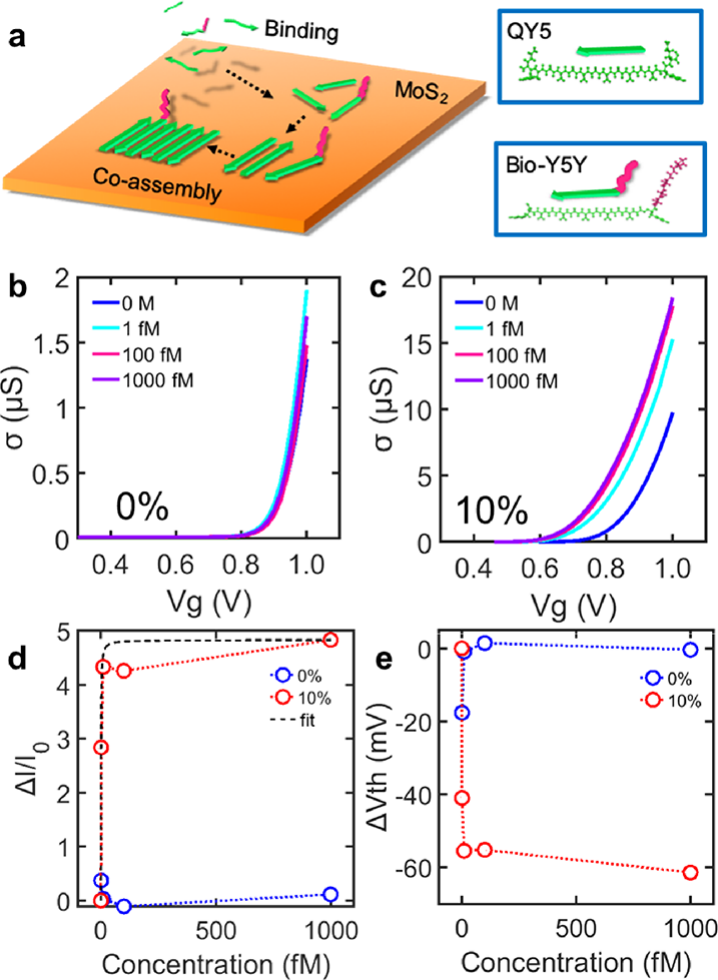
(a) Schematic of peptide co-assembly process with QY5 and Bio-Y5Y on the MoS_2_ surface. The green part of peptides indicates the assembly domain in the sequence, and the red part is its probe that binds to a target molecule. (b) Conductivity of MoS_2_ FETs over applied gate voltages measured at various concentrations of streptavidin. 0% means that the surface was covered by only QY5. (c) Conductivity of MoS_2_ FETs functionalized with the co-assembly of peptides QY5 and Bio-Y5Y with a mixing ratio of 10%. (d) Current changes in devices depending on the concentrations of streptavidin. The fitting curve was plotted based on the Langmuir model. (e) Change of the threshold voltage depending on the streptavidin concentration.

Next, we demonstrated a MoS_2_ biosensor functionalized with self-assembled peptides. Here, we used streptavidin (SA) and biotin for a demonstration of biomolecular detection, which is widely used because of its strong binding affinity. For the immobilization of biotins on MoS_2_, two kinds of peptides were co-assembled^33,34^ as depicted in Figure 4a. In the co-assembly process, we utilized a probe peptide, biotinylated Y5Y (Bio-Y5Y), and a scaffold peptide, QY5 together. We used QY5 as the scaffold peptide because it displayed the most stable behavior in the above experiments. As shown in Table 1, the SSS in the sequence of Bio-Y5Y was adopted as a spacer so that biotin can be exposed on the top of self-assembled peptides since serine “S” is known to be hydrophilic and flexible. Because both peptides share the same sequence of the GA-repeated domain, we expected that they would be self-assembled into a β-sheet-like structure in a mixed form. We defined a mixing ratio as a fraction of Bio-Y5Y concentration over the total concentration of all peptides (Bio-Y5Y + QY5), and the total concentration of peptides was set to be 1 μM. After characterizing various mixing ratios, we utilized here the mixing ratio of 10% because it has a uniform morphology on the MoS_2_ surface with high coverage (Figure S7).

Conductivity measurements at various gate voltages were performed before and after placing a solution of SA with concentrations of 1, 10, 100, and 1000 fM adjusted by 10 mM PB. We measured source-drain currents in the vicinity of *V_th_* for each concentration of SA and obtained Δ*I*/*I*_0_ over concentrations of SA (Figure 4b,c) where *I*_0_ is the current before adding SA. A large change in the current was detected in the case of co-assembled peptides with 10% Bio-Y5Y (Figure 4c). Surprisingly, the 10%-Bio-Y5Y modification on the MoS_2_ biosensor enables 1 fM-level detection. On the other hand, no change was observed by only QY5 without Bio-Y5Y (Figure 4b). The no change indicates that SA has no specific interaction with QY5 on the MoS_2_ surface. The binding event of SA was also confirmed with in situ AFM (Figure S8). Figure 4e shows changes of *V_th_* at each SA concentration. The sample with bioprobes exhibited a clear response to SA in the same way as Figure 4d. It indicates that the co-assembled peptides did not degrade the transconductance of the MoS_2_ FET, and the detection of the SA was made mainly from the shift of *V_th_*. We also demonstrated real-time monitoring of SA with various concentrations (Figure S9), which shows a similar tendency to Figure 4d. The detection mechanism of SA is probably due to a change of charge distribution in MoS_2_ caused by the binding of SA to biotinylated peptides. We believe that the bound SA can change the distribution of electrolyte ions at the vicinity of the MoS_2_ surface and the conformation of the assembled peptides. These events cause the modulation of the polarization at the surface of MoS_2_, resulting in the conductivity change of MoS_2_ FETs.

## 4 Conclusions

In this article, we designed a series of new peptides with the ability to self-assemble into a uniform monomolecular thick layer on MoS_2_ and investigated the impacts of peptides on the electrical property of transistor-based MoS_2_ biosensors. Our peptides revealed sufficient stability as a molecular scaffold for biosensing. Peptides on MoS_2_ did not affect the intrinsic electrical properties of MoS_2_, maintaining a high transconductance of MoS_2_ FETs. On the other hand, the PL of MoS_2_ was sensitively modulated by peptides depending on their amino acid sequences. We succeeded in demonstrating biosensing with biotin and streptavidin, where biotin was immobilized via the co-assembly of biotinylated peptides and scaffold peptides designed in this work. The minimal concentration of streptavidin we successfully detected was 1 fM. This high sensitivity probably arose from the self-assembled peptides, which have a thinner thickness than Debye length. Finally, we note that the usage of self-assembled peptides as a biomolecular scaffold for MoS_2_ biosensors is quite general for any surface functionalization of MoS_2_ toward bioelectronic and biomedical applications.

## Supporting Information Available

Details of peptide synthesis and discussion on morphology of self-assembled peptides characterized by AFM, device fabrication of MoS_2_ FETs, characterization of MoS_2_ FETs, photoluminescence measurements, co-assembly of peptides, and real-time detection of streptavidin by MoS_2_ biosensor (PDF)
