# Supplementary material for: Self-assembled GA-Repeated Peptides as a Biomolecular Scaffold for Biosensing with MoS2 Electrochemical Transistors
Source: ACS Appl Mater Interfaces. 2023 Mar 9;15(11):14058–66. doi: 10.1021/acsami.2c23227 (PMC10037235; doi:10.1021/acsami.2c23227)
Supplement: Supplementary file 1 — am2c23227_si_001.pdf [file am2c23227_si_001.pdf]

Supporting Information on

Self-assembled GA-repeated peptides as a biomolecular scaffold for biosensing with MoS_2 electrochemical transistors

Hironaga Noguchi[1], Yoshiki Nakamura[1], Sayaka Tezuka[1],

Takakazu Seki[2,], Kazuki Yatsu[1], Takuma Narimatsu[1], Yasuaki Nakata[1], and Yuhei Hayamizu[1,*]*

1. Department of Materials Science and Engineering, School of Materials and Chemical Technology, Tokyo Institute of Technology, Tokyo 152-8550, Japan

2. Department of Frontier Materials Chemistry, Faculty of Science and Technology, Hirosaki University, 3 Bunkyo-Cho, Hirosaki-shi, Aomori 036-8561, Japan

Table of Contents

1: Peptide Synthesis

2: Morphology of Self-assembled Peptides Characterized by AFM

3: Device Fabrication of MoS_2 FET

4: Characterization of MoS_2 FET

5: Photoluminescence measurements

6: Co-assembly of peptides

7: Realtime detection of streptavidin by MoS_2 biosensor

1: Peptide Synthesis

In this work, peptides were synthesized by a solid phase peptide synthesis method[1] (see the peptide sequence in Fig. S1). Rink Amide MBHA resin (0.1 mmol, 100−200 mesh, Merck Millipore, JP) as a solid support in peptide synthesis was swelled in 2 mL N,N-dimethylformamide (DMF, SP Grade 99.9%, Watanabe Chemical Industries, LTD. JP) overnight. Then, the resin was deprotected using 2 mL 20% piperidine (99%, Nacalai Tesque, Inc. JP) for 10 minutes at room temperature and washed with DMF. For peptide extension, we utilized 1-hydroxybenzotriazole monohydrate (HOBT· H_2O, TCI CO., LTD. JP), O-(benzotriazol-1-yl)-N,N,N',N'-tetramethyluronium hexafluorophosphate (HBTU, Watanabe Chemical Industries, LTD. JP) and N,N-diisopropylethylamine (DIEA, Watanabe Chemical Industries, LTD. JP) as coupling reagents, and 700 μL of 0.45 M HBTU/HOBT in DMF and 0.9 M DIEA in DMF and 0.3 mmol N-terminal-protected amino acid (Watanabe Chemical Industries, LTD. JP) protected by 9-fluorenylmethyloxycarbonyl group (Fmoc) were added into resin solution. The reaction mixture was stirred for 15 minutes at room temperature. After the reaction, DMF and dichloromethane (DCM, Watanabe Chemical Industries, LTD. JP) were utilized to wash resin and remove impurity. Cycles of deprotection-wash-coupling-wash were repeated. Then, we obtained the resin with target peptides, which was washed with methanol and dried in a vacuum desiccator. The target peptides were cleaved from the resin with 1.5 mL trifluoroacetic acid (TFA, TCI CO., LTD. JP)/water (Direct-Q UV3, Merck Millipore, JP)/triisopropylsilane (TIPS, Watanabe Chemical Industries, LTD. JP) (volumetric ratio: TFA/water/TIPS = 2350:62.5:62.5) for 2 hours at room temperature. Peptides dissolved in solution were extracted by adding 12 mL diethyl ether (SHOWA-ETHER, JP). Since solubility of peptide in ether is usually much lower than that in TFA, peptide precipitates upon addition of "ether". The precipitate of peptide in ether was collected with centrifugation. After removing supernatant, we further washed peptide precipitate with "ether" twice. Subsequently, the collected peptide precipitate was dried up in a vacuum desiccator. The obtained crude peptide powder was purified by a reverse-phase HPLC using acetonitrile/water mobile phase (Waters 2489 UV/ Visible detector, Waters 1525 Binary HPLC pump, Waters Corporation, JP) in a C18 column (COSMOSIL Packed Column 5C 18-AR-II, 20 ID × 250 mm, Nacalai Tesque, Inc. JP). The obtained peptides were analyzed by MALDI-TOF-MS (Shimadzu Axima-performance, 337 nm nitrogen laser, 20 kV, Shimadzu, JP) using α- cyano-4-hydroxycinnamic acid (98.0%, TCI CO., LTD. JP) as a matrix.

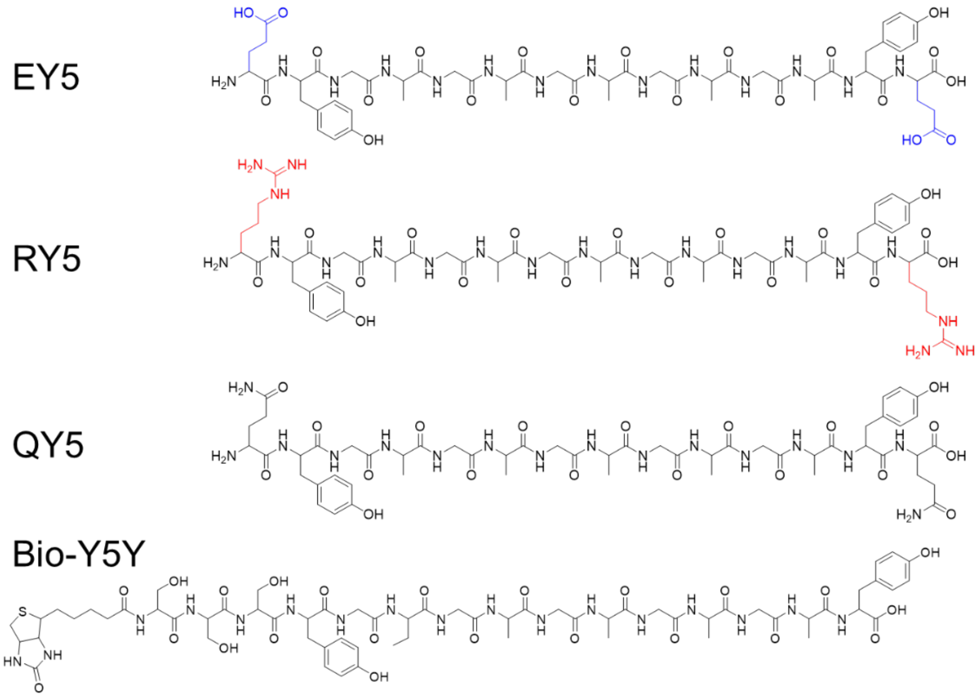

Figure S1. Molecular structures of peptides: EY5, RY5, QY5, and Bio-Y5Y.

2: Morphology of Self-assembled Peptides Characterized by AFM

We compare the self-assembled structures incubated at various concentrations of peptide solutions (Fig. S2). The surface coverage increased with increasing concentration of peptide solution. The fast Fourier transform (FFT) image shows that all peptides have a hexagonal symmetrical structure. As shown in Fig. 1d, EY5 has the smallest binding affinity to the MoS_2 surface. EY5 does not exhibit nanowire structures clearly even at 100 nM. On the other hand, RY5 and QY5 have linear wire structures at the same concentration.

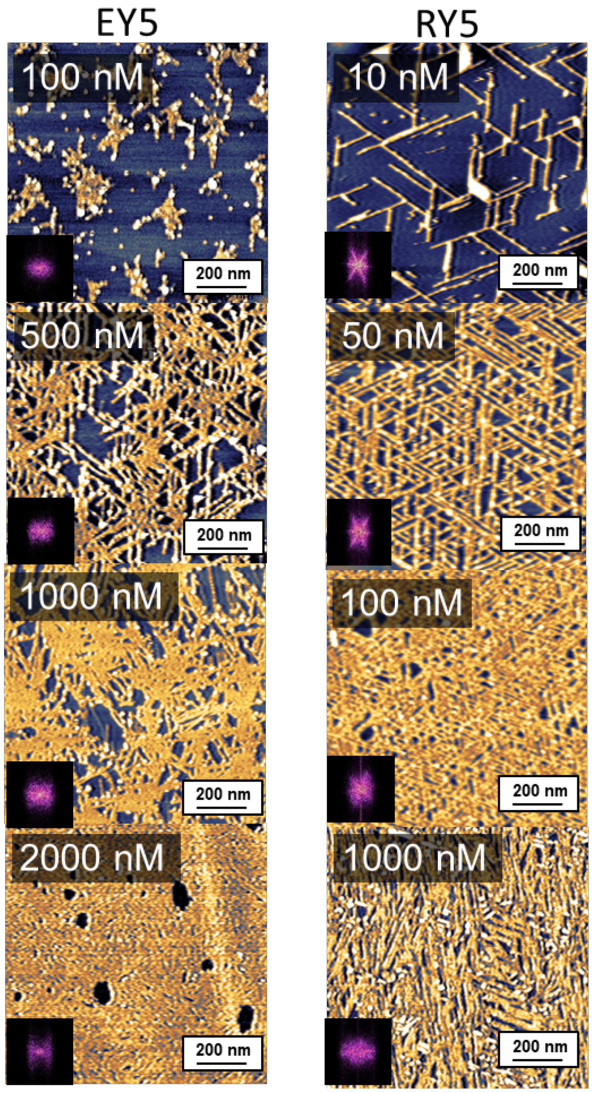

Figure S2. AFM images of self-assembled peptides on MoS_2 at different concentrations. The insets are fast Fourier transform images of the corresponding AFM images. All peptides showed a six-fold symmetric feature clearly, suggesting that the peptides recognized the lattice of the underlying MoS_2 substrate.

3: Device Fabrication of MoS_2 FET

The MoS_2 field effect transistors (FETs) were fabricated as following steps. First, electrodes were fabricated with a standard photolithography technique by depositing 10-nm Ti and 40-nm Au in a vacuum thermal evaporator. MoS_2 was grown on a Si substrate by chemical vapor deposition. Then, the MoS_2 were transferred on a Si wafer (with 270-nm thick SiO_2 layer) with patterned electrodes. In this process, polystyrene (PS) was used as a supporting film and it was transferred

by a Poly(dimethylsiloxane) PDMS stamp[2]. The transferred PS supporting film was removed by immersing in toluene at 90 °C for 30 minutes. Sequentially, the substrate was rinsed with toluene at room temperature to remove the polymer residues completely at room temperature, and it was dried with nitrogen blow. As the result of this process, the CVD-grown MoS_2 was placed on desired electrodes to form a FET. As a protection layer for electrodes, polymethyl methacrylate (PMMA) was deposited on electrodes with a toothpick by hands. The procedure is summarized in Figs. S3a and S3b.

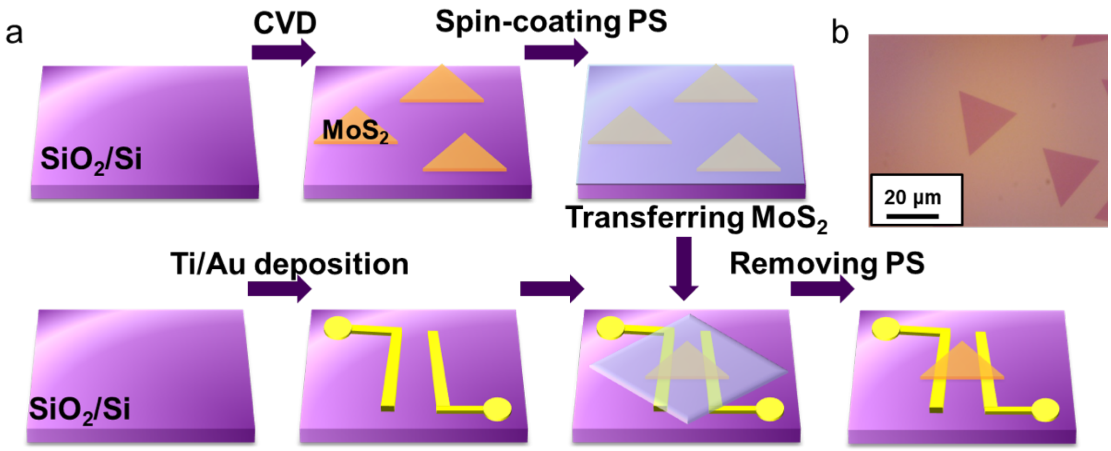

Figure S3. (**a**) Schematics of fabrication process. (**b**) Optical microscope image of CVD-grown MoS_2 on a Si wafer substrate.

The number of layers of MoS_2 was evaluated by optical spectroscopy. It was reported that single-layer MoS_2 shows a strong photoluminescence (PL) due to its direct band-gap semiconducting property[3]. Figs. S4a and S4b show the optical image and PL image of the prepared MoS_2 FET. Our MoS_2 in FETs showed a bright and uniform PL, indicating its direct band-gap property. Raman spectrum of the MoS_2 reveals two peaks at 383.7 cm^{-1} and 404.7 cm^{-1} as shown in Fig.S4c. It is well known that these peaks correspond to the vibrational modes of E_{2g} and A_{1g}, respectively, and MoS_2 has a single layer when the position difference between the peaks is 20 cm^{-1} or less[4,5]. In this work, the difference of the peak position was 18.7 cm^{-1}. These observations support that MoS_2 in FETs in this work consists of single layer.

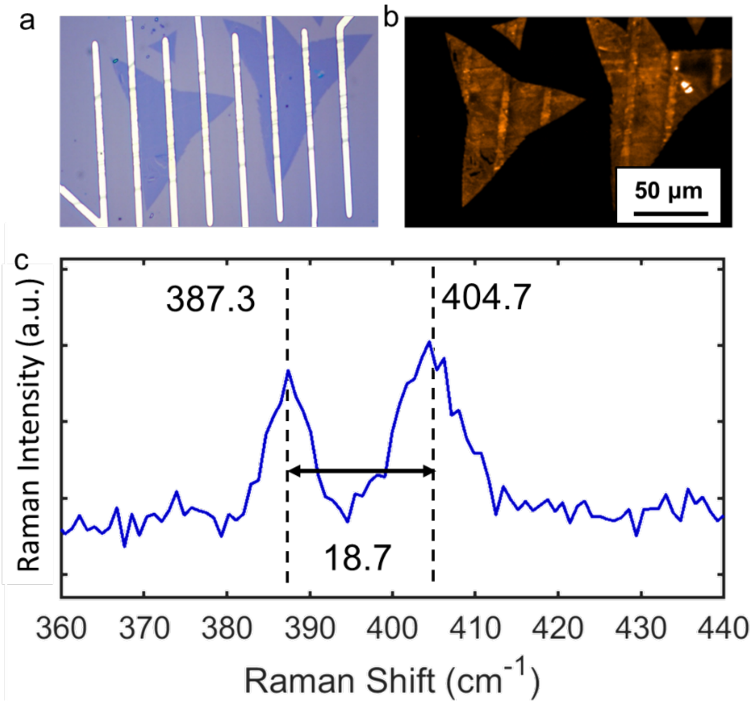

Figure S4. (**a**) Optical microscope image of transferred MoS_2 on patterned electrodes. (**b**) PL image of **a** under photoexcitation. (**c**) Raman spectrum of MoS_2 used for a FET.

4: Characterization of MoS_2 FET

Fig. S5a shows a linearity of source-drain current over applied source-drain voltage, indicating its ohmic nature. We utilized $V_{sd} = 30$ mV in all the measurement for gate response of FETs, which is in the range of the linear region. Gate response of source-drain current is shown in Fig. 2c as a typical example. We analyzed these gate responses for all the FETs. Here, the gate voltage was swept from 0.3 V to 1.0 V, cyclically. The threshold voltage was estimated by the fit with a linear function, where the linear function was fitted in the linear region of the source-drain current. We found that the current shows a small hysteresis. We also evaluated the threshold for both forward and backward sweeps in the same manner. We defined the hysteresis of the threshold voltage as the difference of these threshold voltages. Furthermore, we also derived a transconductance g_m of FETs, which was obtained from a linear fitting as well. In Figs. S5b-d, we evaluated a uniformity of device properties in this work. Figs. S5b and d show that the distributions of threshold voltage and transconductance are relatively narrow, indicating that the device fabrication was successfully done without significant fluctuations. On the other hand, the hysteresis of the threshold voltage shows a relatively large distribution (Fig. S5c). It could be related to the surface condition of MoS_2. We characterized at least three devices for each peptide

at each incubation condition to derive error bar (Figs. 2f-h). The total number of devices we characterized in this work was 40.

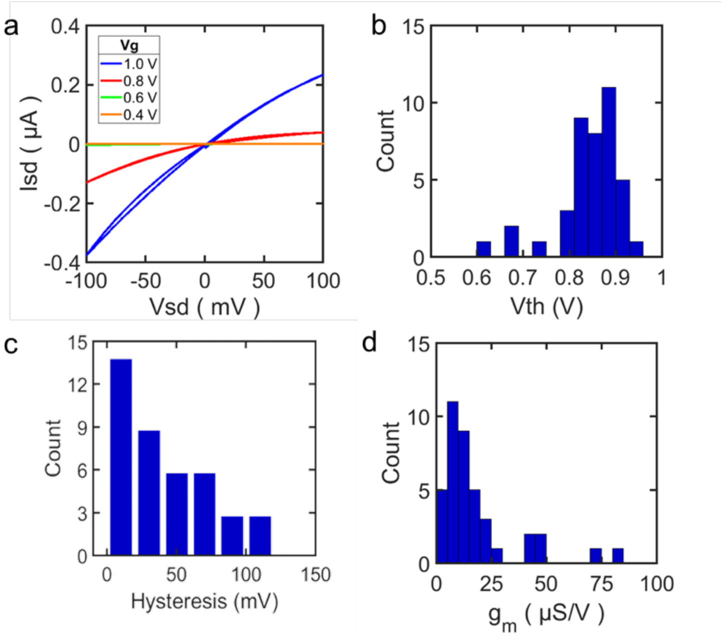

Figure S5. (**a**) Electrical conductivity of MoS_2 FET under various gate voltages V_g. A plot of source-drain current vs. source-drain voltage. (**b**) Histogram of threshold voltage of all the MoS_2 FETs used in this work. (**c**) Histogram of hysteresis of threshold voltage during the cyclic sweeping of gate voltage. (**d**) Histogram of transconductance g_m of all FETs.

5: PL measurements

The PL of single-layer MoS_2 was obtained by an inverted microscope (Olympus IX73) with a spectrometer (Oxford instruments, Shamrock 193i) equipped with an electron multiplying charge coupled device (Oxford instruments, iXon-Ultra 888 EMCCD). The excitation light from a mercury lamp was guided to a sample through a band pass filter, a dichroic mirror, and 100X objective lens (N.A = 0.95). The 546-nm line in the mercury lamp was used for the excitation. The spectrometer contains gratings and a mirror. These gratings and mirror are switchable. The mirror allows us to obtain PL images by fully opening an optical slit in the spectrometer (Fig. S6a). Alternatively, the grating and optical slit allows us to measure spectrograph containing spatial information (Fig. S6b). This image shows local PL from the CVD-MoS_2, which allows us

to see the spatial distribution of PL spectra over the single-layer MoS_2. A typical PL spectrum is shown in Fig. S6c. The PL peak position and intensity varied at each location. It is probably due to the internal strain and cracks in the MoS_2. The peak positions and intensity at each location were collected, and then the difference between before and after peptide assembly was calculated (Fig. 3).

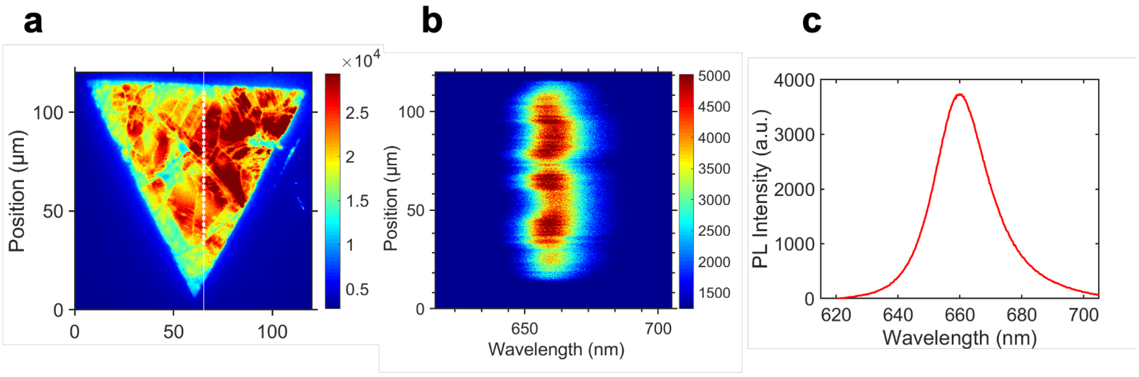

Figure S6. (**a**) A PL image of single-layer CVD-MoS_2 under photoexcitation. (**b**) Corresponding spectrograph of the MoS_2. While x axis shows the wavelength of PL, y axis shows the position dependence of PL indicated as the dashed white line in (**a**). The color bar indicates the PL intensity (counts). (**c**) photoluminescence spectrum obtained by averaging the PL spectra at the locations indicated by the white dashed line in (**a**).

7: Co-assembly of peptides

We defined the mixing ratio as a fraction of Bio-Y5Y concentration over total concentration of all peptides (Bio-Y5Y + QY5). All the sample showed a high coverage (Fig. S7). It indicates that the mixing of Bio-Y5Y did not affect their binding affinity to the MoS_2 surface. In AFM images, some bare areas with round shape were found. It could be caused by air bubble at the interface when we incubated the peptide solution on the MoS_2 surface. The thickness of monomolecular structures is uniform, indicating that Bio-Y5Y and QY5 are miscible each other when they self-assembled on surface.

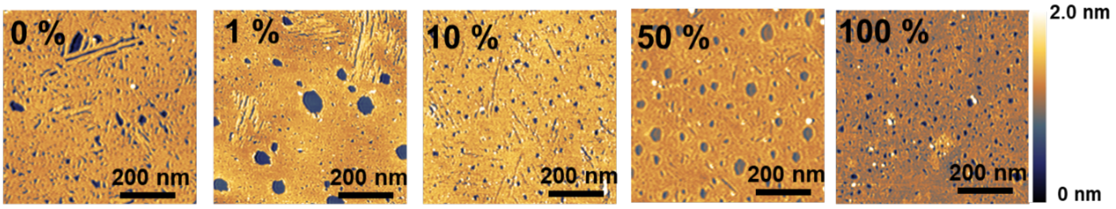

Figure S7. AFM images showing the morphology of co-assembly of Bio-Y5Y/ QY5 with various mixing ratios.

We monitored the morphology of co-assembled peptides at the same location by in-situ AFM while we sequentially added streptavidin (SA) with different concentrations (Fig. S8). Before adding SA, we observed fine linear structures of peptides. It manifests that the co-assembled peptide forms long-range ordered structures even after mixing two kinds of peptides, Bio-Y5Y and QY5. It indicates that these peptides are miscible, probably because they shared the GA domain for their interpeptide interactions. After adding SA, we found that there are small dots with slightly bright color in height images increased as we increased the concentration of SA. This tendency was similarly observed in phase images. It is perhaps because of binding of SA to biotin in the co-assembled peptides. The tendency of SA binding is consistent with our finding in the electrical measurements (Fig. 4).

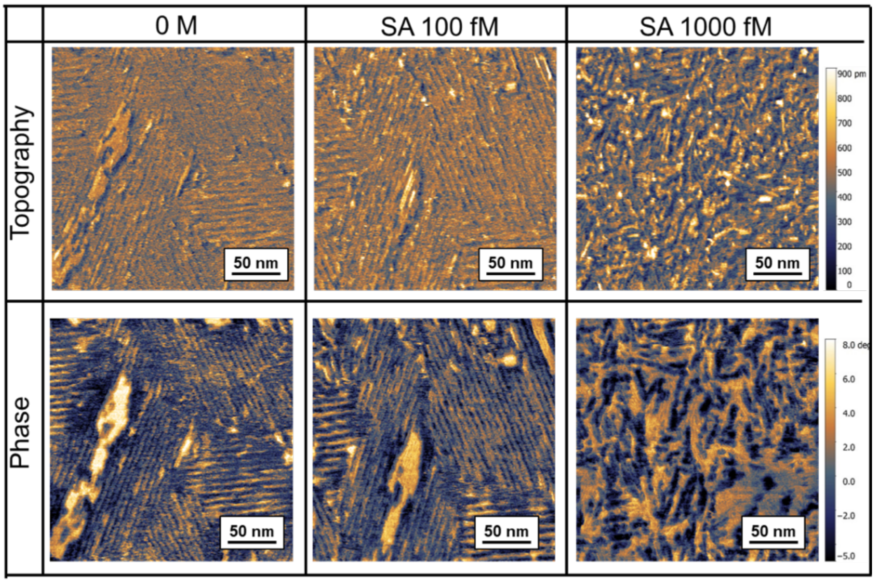

Figure S8. In situ AFM images of adsorbed streptavidin on Bio-Y5Y/QY5 co-assembled structure at the mixing ratio of 10%. in-situ AFM topography and phase images showing the adsorption of streptavidin at different concentrations of 100 fM and 1000 fM.

8: Real time detection of streptavidin by MoS_2 biosensor

The source-drain current was measured by fixing the gate voltage and the source drain voltage constant at 0.5 V and 30 mV, respectively. After incubation of peptide with 50% mixing ratio, we waited for the source-drain current stabilized. Before adding SA in the solution, we replaced the solution with a 10-mM PB solution using a pipette several times to remove excess peptides. After waiting for about 20 min, SA solutions with 10-mM PB were added every 10 minutes to characterize the response of MoS_2 FET against the SA. The concentrations of SA were increased from 1 fM to 100 nM. Fig.S9a shows the change of source-drain current over time. The current decreased with increasing the SA concentration. The average value of the current at each concentration was derived, and the change of current was plotted against the SA concentration (Fig. S9b). The change of the current was saturated at 10 fM–1 pM. It indicates that the binding affinity of SA to biotin estimated by our MoS_2 biosensor was in the range of 10 fM or less. At even higher concentrations, the current change increased again. This may be strongly influenced by the multilayer adsorption of SA on the MoS_2 surface. A change of dielectric constant of the solution due to the increasing concentration of SA could be another reason of the current change at the high concentration region. Thus, we fitted the current change in the range of 1–1000 fM with the Langmuir model, and obtained a binding affinity of 1 fM. This value is similar to the one we obtained in Fig. 4d.

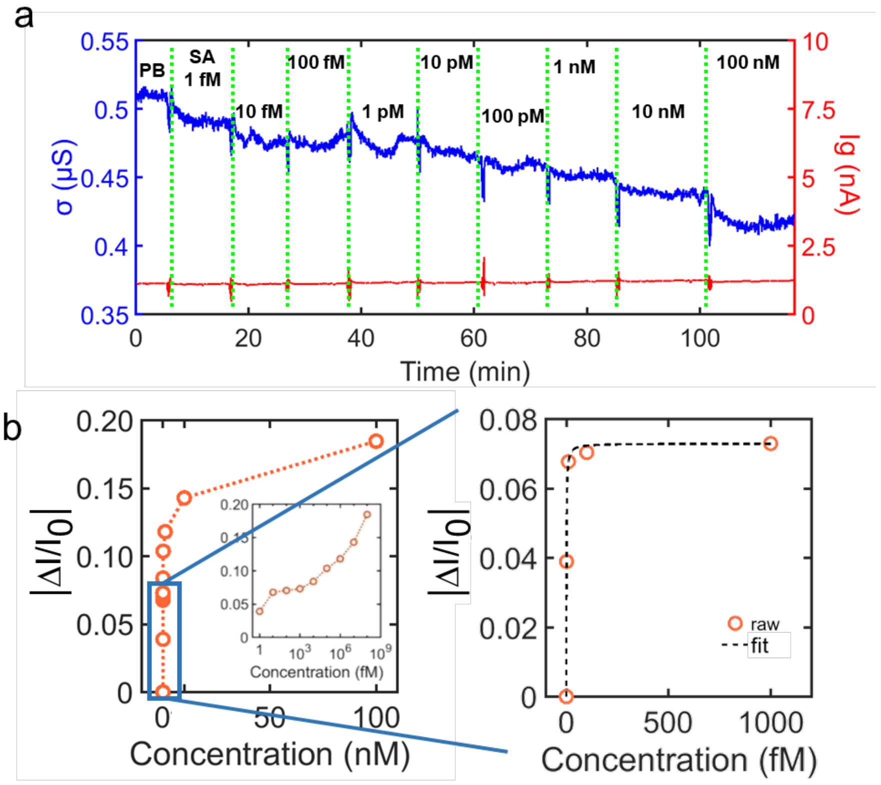

Figure S9. (**a**) A plot of conductivity of MoS_2 FET vs time. We sequentially added SA solution with different concentrations into the test solution to monitor the electrical response of the MoS_2 FET. (**b**) Current change vs concentration of SA. The fitting curve was made with Langmuir model. The inset shows a same plot with the x-axis with logarithmic scale.

Supplementary References

(1) Merrifield, R. B. Solid Phase Peptide Synthesis. I. The Synthesis of a Tetrapeptide. *J. Am. Chem. Soc.* **1963**, *85*, 2149–2154.

(2) Gurarslan, A.; Yu, Y.; Su, L.; Yu, Y.; Suarez, F.; Yao, S.; Zhu, Y.; Ozturk, M.; Zhang, Y.; Cao, L. Surface-Energy-Assisted Perfect Transfer of Centimeter-Scale Monolayer and Few-Layer MoS_2 Films onto Arbitrary Substrates. *ACS Nano* **2014**, *8*, 11522–11528.

(3) Mak, K. F.; Lee, C.; Hone, J.; Shan, J.; Heinz, T. F. Atomically Thin MoS2: A New Direct-Gap Semiconductor. *Phys. Rev. Lett.* **2010**, *105*, 136805.

(4) Lee, C.; Yan, H.; Brus, L. E.; Heinz, T. F.; Hone, J.; Ryu, S. Anomalous Lattice Vibrations of Single- and Few-Layer MoS2. *ACS Nano* **2010**, *4*, 2695–2700.

(5) Li, H.; Zhang, Q.; Yap, C. C. R.; Tay, B. K.; Edwin, T. H. T.; Olivier, A.; Baillargeat, D. From Bulk to Monolayer MoS 2: Evolution of Raman Scattering. *Adv. Funct. Mater.* **2012**, *22*, 1385–1390.
